# Promotion of health care workers immunization in Europe – the main objective of the HProImmune project

**DOI:** 10.1186/1471-2334-13-S1-O12

**Published:** 2013-12-16

**Authors:** Daniela Pițigoi, Liliana Lucia Preoțescu, Anca Streinu-Cercel, Alexandru Rafila, Adrian Streinu-Cercel

**Affiliations:** 1 Carol Davila University of Medicine and Pharmacy, Bucharest, Romania; 2 National Institute for Infectious Diseases “Prof. Dr. Matei Balş”, Bucharest, Romania

## Background

The National Institute for Infectious Diseases “Prof. Dr. Matei Balş” is participating during 2012-2014 in the European project “HProImmune - Promotion of immunization among health care workers in Europe”, coordinated by the Institute of Preventive Medicine, Environmental and Occupational Health, Prolepsis from Greece and co-funded by DG SANCO Public Health Work Program 2008-2013.

The general objective of this project is to promote vaccination coverage of health care workers (HCWs) in different health care settings by developing a tailored communication toolkit.

## Methods

In the first part of the project each partner reviewed and summarized the existing information and best practices regarding the immunization of HCWs in Europe and explored behaviors and barriers regarding HCWs immunization through qualitative analysis (focus groups), that will contribute to the development in the second part of the project of a toolkit for the promotion of immunization among HCWs in Europe.

## Results

The following goals have already been accomplished:

- a list of priority vaccine preventable diseases posing major threat to the health of HCWs: hepatitis B, influenza, measles, mumps, rubella, tetanus, diphtheria, pertussis and varicella.

- a database with information on vaccination coverage, recommendations, guidelines, immunization policies and the legal framework regarding the vaccination of HCWs around Europe

- each country organized focus groups with different stakeholders to understand the risk perception, behaviors towards vaccination and barriers inhibiting HCWs from immunization.

## Conclusion

Increased awareness through training and knowledge provision is expected to enable HCWs to better protect their health and act as role models for their workplace and community.

